# Protective Role of *Helicobacter pylori* Infection in Prognosis of Gastric Cancer: Evidence from 2454 Patients with Gastric Cancer

**DOI:** 10.1371/journal.pone.0062440

**Published:** 2013-05-07

**Authors:** Fang Wang, Guoping Sun, Yanfeng Zou, Fei Zhong, Tai Ma, Xiaoqiu Li

**Affiliations:** 1 Department of Oncology, The First Affiliated Hospital of Anhui Medical University, Hefei, Anhui, China; 2 Department of Epidemiology and Biostatistics, School of Public Health, Anhui Medical University, Hefei, Anhui, China; University of Aberdeen, United Kingdom

## Abstract

**Background:**

A number of studies have investigated the association between *Helicobacter pylori* (*H. pylori*) infection and the prognosis of gastric cancer (GC), with inconsistent and inconclusive results. We performed a meta-analysis to derive a more precise estimation of the association.

**Methodology/Principal Findings:**

A systematic search of PubMed, EMBASE, Cochrane and Chinese wanfang databases was performed with the last search updated on February 19, 2013. The hazard ratio (HR) and its 95% confidence interval (95%CI) were used to assess the strength of association. A total of 12 studies including 2454 patients with GC were involved in this meta-analysis. The pooled HR was 0.71 (95%CI: 0.57–0.87; *P* = 0.001) for OS and 0.60 (95%CI: 0.30–1.18; *P* = 0.139) for DFS in GC patients, respectively. The protective role of *H. pylori* infection in the prognosis of GC was also observed among different subgroups stratified by ethnicity, statistical methodology, *H. pylori* evaluation method and quality assessment. There was no evidence of publication bias.

**Conclusions/Significance:**

This meta-analysis suggests a protective role for *H. pylori* infection in the prognosis of GC. The underlying mechanisms need to be further elucidated, which could provide new therapeutic approaches for GC.

## Introduction

Gastric cancer (GC) remains the fourth most common malignancy and the second most common cause of cancer-related deaths throughout the world [1]. Despite recent advances in surgical techniques combined with neoadjuvant chemotherapy and radiotherapy approaches, patients with advanced disease still have a poor outlook [2]. Most cases have locally advanced disease when diagnosed, with a 5-year survival rate of only 20% to 25% [3]. In the era of personalized medicine, it is necessary to find prognostic and predictive factors that can be used to modify treatment strategies.

*Helicobacter pylori* (*H. pylori*) is a Gram-negative, microaerophilic bacterium which is the major causative agent of gastritis, peptic ulcer disease, mucosa-associated lymphoid tissue (MALT) lymphoma, and GC [4]. Moreover, International Agency for Research on Cancer categorized *H. pylori* as a group 1 carcinogen for GC in 1994 [5]. To date, an increasing body of evidence indicates that *H. pylori* infection increases the risk of developing adenocarcinoma of the distal stomach [6]–[7]. Meanwhile, some researchers have focused on the association between *H. pylori* status and the prognosis of GC patients [8]–[19]. Several studies suggested that patients with GC who are negative for *H. pylori* have a poor outlook than those positive [9]–[11], [16], [18]. However, some other studies did not provide evidence of a better prognosis in patients with *H. pylori* infection compared with negative subjects [8], [12]–[15], [17], [19].

These reported results were inconsistent and conflicting with no clear consensus. Therefore, we performed a meta-analysis to derive a more precise estimation of the association between *H. pylori* infection and the prognosis of GC.

## Materials and Methods

### Identification of Studies

We conducted a comprehensive search of medical literature on studies evaluating the effect of *H. pylori* infection on the prognosis of GC. We searched the US National Library of Medicine’s PubMed database, Excerpta Medica Database (EMBASE), the Cochrane Central Register of Controlled Trials and Chinese wanfang database using the keywords“*Helicobacter pylori*”, “*H. pylori*”, “gastric cancer”, “gastric carcinoma”, “prognosis”, “survival”, “recurrence”, and “relapse” with the last search updated on February 19, 2013. There is no restriction on language or publication years in the selection process. All of the references from review papers and original reports were checked for further relevant studies in the systematic review. Search was performed independently by two reviewers (WF and ZYF), and disagreement was resolved by discussion with our research team.

### Eligibility Criteria

Studies were eligible if survival was analyzed in GC patients stratified by *H. pylori* status. The primary outcome of interest was overall survival (OS). The secondary outcome of interest was disease-free survival (DFS). Criteria for eligibility of a study to the present meta-analysis were: to present a proven diagnosis of GC in humans; to evaluate the association between *H. pylori* status and patient survival; to provide hazard ratios (HRs) with its corresponding 95% confidence intervals (CIs) or sufficient data for estimating HR with 95%CI.

### Data Extraction

Data extraction was performed independently by two reviewers (WF and ZF). Disagreement was resolved by discussion with our research team. For each study the following information were collected: the first author’s name, ethnicity, year of publication, definition of cases, sample size, *H. pylori* evaluation method, number of patients with positive *H. pylori* status and prognostic information. If the required information were unavailable in relevant articles, a request was sent to the corresponding author for additional data. If a study reported the results on different ethnicities, we treated them as separate studies.

### Quality Assessment

Quality assessment was performed with the Newcastle-Ottawa quality assessment scale (NOS) for cohort studies. Each study was judged on three broad perspectives: selection, comparability and outcome. The maximum score was 9 and a high-quality study was defined as one with a score of ≥6. Quality assessment was performed independently by two reviewers (MT and LXQ), and disagreement was resolved by discussion with our research team.

### Statistical Analysis

We used the PRISMA checklist as protocol of the meta-analysis and followed the guideline (Table S1) [20]. The HR and its 95%CI were used to assess the strength of association. Heterogeneity among studies was assessed by using the chi-square test, expressed with the Q-statistic and *I^2^* statistic, as described by Higgins and colleagues [21]. *I^2^* was measured from 0–100% with increasing *I^2^* values indicating a larger impact of between-study heterogeneity in the meta-analysis. When substantial heterogeneity was detected, the summary estimate based on the random effects model (DerSimonian and Laird method) was reported [22]. Otherwise, the summary estimate based on the fixed effects model (the inverse variance method) was reported [23].

The most accurate method comprised of retrieving the HR estimate and its 95%CI from the reported results, or calculating them from the presented data using two of the following parameters: the HR point estimate, the log-rank statistic or its *P* value, the O–E statistic or its variance [24]. In those studies where only survival curve was available, the survival curve was used to reconstruct HR and its variance, with the assumption that patient censor rate was constant during study follow-up. This method has been described by Parmar and colleagues [25]. All data analyses were carried out using *H. pylori* negative group as the reference group (HR = 1). An observed HR of >1 implied a worse survival for patients with positive *H. pylori* status. In the study by Kurtenkov et al. [9], separate HR estimates according to different stages (stage I and II) were reported. However, the study did not report the effect of combined stages. In this situation, the study-specific effect size in overall analysis was recalculated by pooling the HR estimated of different stages by using the inverse-variance method.

We used Egger’s test (linear regression method) [26] and Begg’s test (rank correlation method) [27] to evaluate the potential publication bias. *P*<0.05 for Egger’s or Begg’s test was considered to be representative of significant statistical publication bias. All statistical analyses were undertaken using Stata version 10 (StataCorp LP, College Station, Texas, USA). Kaplan-Meier curves were read by Engauge Digitizer version 4.1 (http://digitizer.sourceforge.net). Statistical tests were two-sided and *P* values less than 0.05 were considered significant.

## Results

### Literature Search

**Figure 1:**
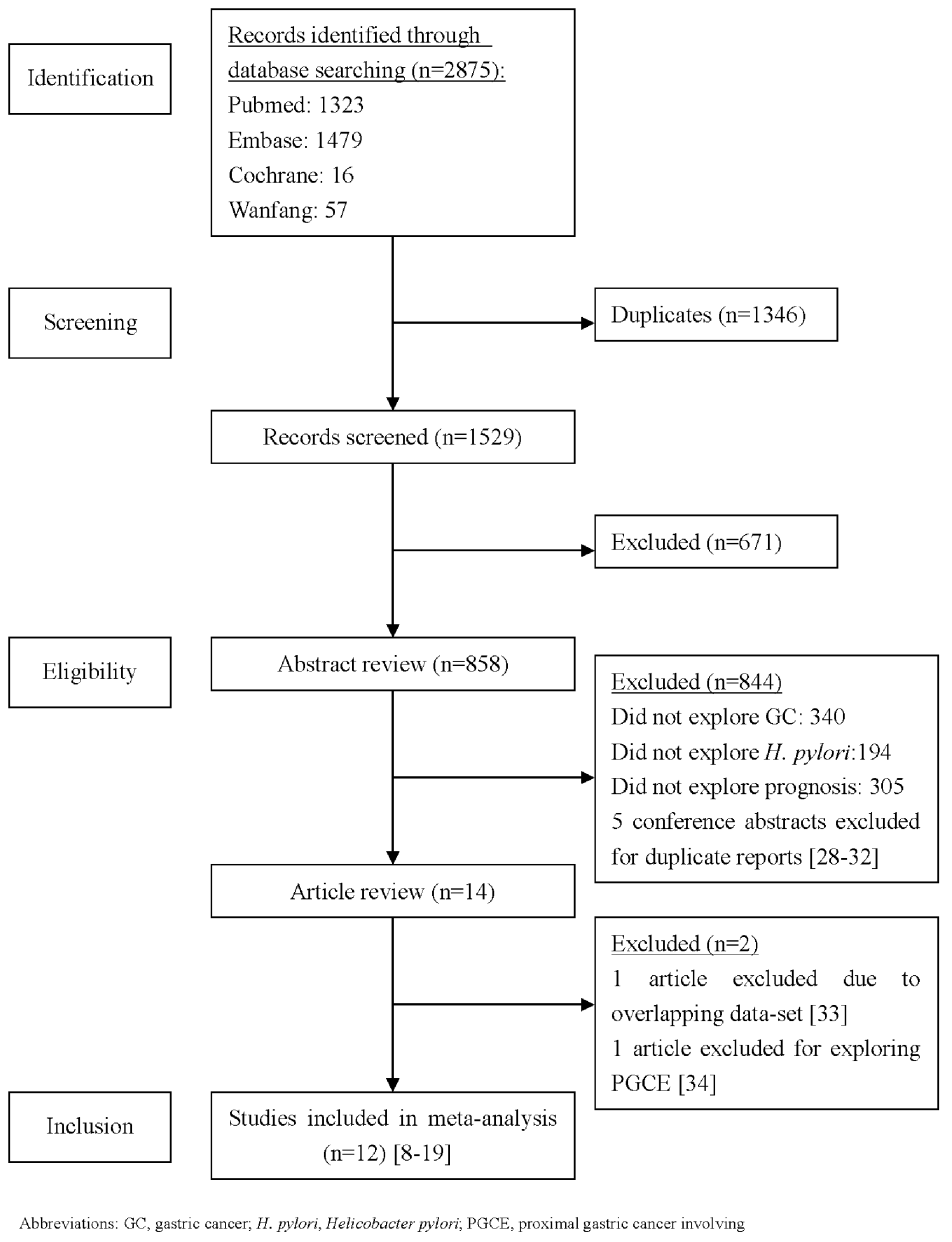
The flow chart of the included studies in the meta-analysis.

Our systematic literature search yielded a total of 12 studies associated with *H. pylori* infection and the prognosis of GC in the final analysis [8]–[19]. Figure 1 illustrates the search process and the final selection of relevant studies. Of the 1529 potential relevant records after duplications removed, 1510 records were excluded after we had reviewed the titles and abstracts. After carefully reviewing the remaining 19 studies [8]–[19], [28]–[34], a total of 12 studies were eligible for the final analysis. Five conference abstracts were excluded for duplicate reports [28]–[32]. There were two studies from the same population, both reported by Lee et al. [8], [33]. Under this circumstance, the study with larger sample size was included [8], while the other study was excluded due to overlapping data-set [33]. The study by Zhang et al. [34] was excluded because it focused on patients with proximal gastric carcinoma involving the esophagus (PGCE).

### Study Characteristics and Quality Assessment

The main characteristics for the studies included in our meta-analysis are summarized in Table 1. Among these studies, 7 studies were performed in Asians [8], [12], [13], [15], [16], [18], [19], 4 studies were performed in Caucasians [9]–[11], [17] and 1 study was performed in Brazilian [14]. Sample sizes ranged from 61 [18], [19] to 794 patients [13], with a total of 2454 GC patients. The positive rate of *H. pylori* varied from 17.5% [15] to 86.2% [11]. *H. pylori* status was evaluated by different methods in these studies, which mainly included serologic detection, histological analysis and polymerase chain reaction (PCR). We were able to extract overall survival (OS) information from all the studies on GC. Nevertheless, we were able to extract disease-free survival (DFS) information from only 3 studies [10], [12], [19].

**Table 1:** Characteristics of studies that evaluated the impact of *H. pylori* infection on the prognosis of gastric cancer.

Study ID	Authors	Year	Ethnicity	Sample Size	Patients positive for*H. pylori* (%)	*H. pylori* Evaluation Method	Prognostic information HRΔ(95%CI)	Quality Score
1	Lee et al. [8]	1995	Asian	151	92(60.9)	Serologic detection	OS:0.91(0.51–1.62)^m^	7/9
2	Kurtenkov et al. [9]	2003	Caucasian	87	NA	Serologic detection	OS:0.74(0.63–0.87)^u^	6/9
3	Meimarakis et al. [10]	2006	Caucasian	166	125(75.3)	Serologic detection, Histological analysis, Bacterial culture	OS:0.50(0.31–0.82)^m^ DFS:0.46(0.29–0.75)^m^	8/9
4	Marrelli et al. [11]	2009	Caucasian	297	256(86.2)	Serologic detection, PCR	OS:0.40(0.23–0.71)^m^	9/9
5	Qiu et al. [12]	2010	Asian	157	82(52.2)	PCR	OS:1.09(0.70–1.68)^u^ DFS:1.13(0.67–1.92)^u^	8/9
6	Gan et al. [13]	2011	Asian	794	239(30.1)	Histological analysis	OS:0.87(0.70–1.08)^m^	8/9
7	Santos et al. [14]	2011	Brazilian	68	34(50.0)	Histological analysis	OS:0.68(0.40–1.16)^m^	8/9
8	Chen et al. [15]	2012	Asian	120	21(17.5)	PCR	OS:1.50(0.75–3.00)^u^	5/9
9	Kang et al. [16]	2012	Asian	274	166(60.6)	Histological analysis	OS:0.29(0.20–0.41)^m^	8/9
10	Syrios et al. [17]	2012	Caucasian	218	76(34.9)	Serologic detection	OS:0.88(0.66–1.16)^u^	7/9
11	Choi et al. [18]	2012	Asian	61	19(31.1)	Histological analysis	OS: 0.78(0.63–0.97)^u^	5/9
12	Hur et al. [19]	2012	Asian	61	40(65.6)	Serologic detection, Histological analysis	OS:0.62(0.25–1.54)^u^ DFS:0.37(0.16–0.84)^m^	7/9

Abbreviations: *H. pylori*, *Helicobacter pylori*; OS, overall survival; DFS, disease-free survival; PCR, Polymerase chain reaction; HR, hazard ratio; CI, confidence interval;

u, univariate result;

m, multivariate result; NA, not available.

Δ HR = 1 for negative *H. pylori* status.

The range of quality scores was from 4 to 9 stars, with a higher value indicating better methodology (see Table S2). Ten studies that had ≥6 awarded stars were categorized as high quality studies [8]–[14], [16], [17], [19], while 2 studies that had <6 awarded stars were categorized as low quality studies [15], [18].

### Overall Analysis

**Figure 2:**
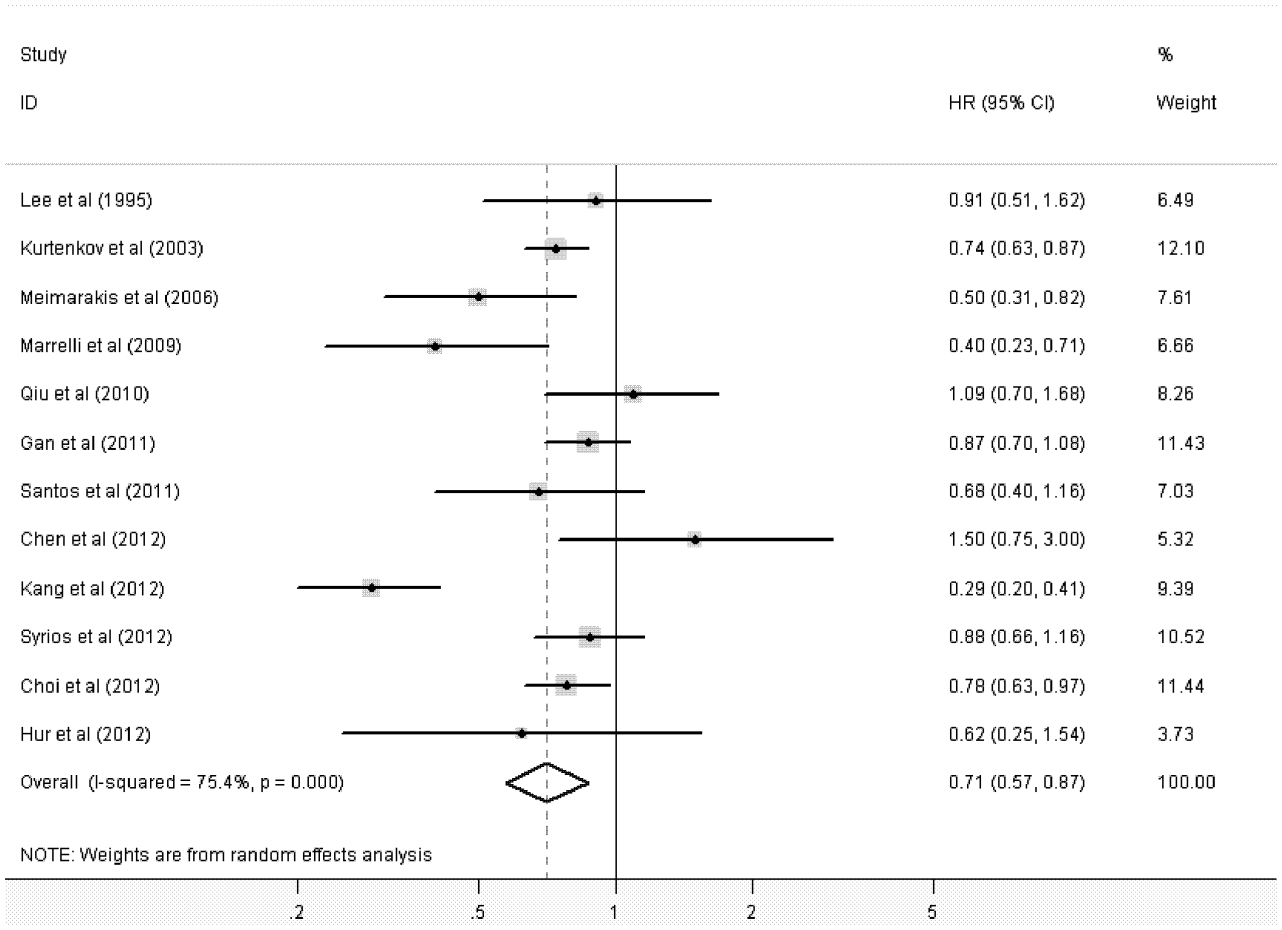
Forest plot showing the meta-analysis of hazard ratios estimates for overall survival in gastric cancer patients.

**Figure 3:**
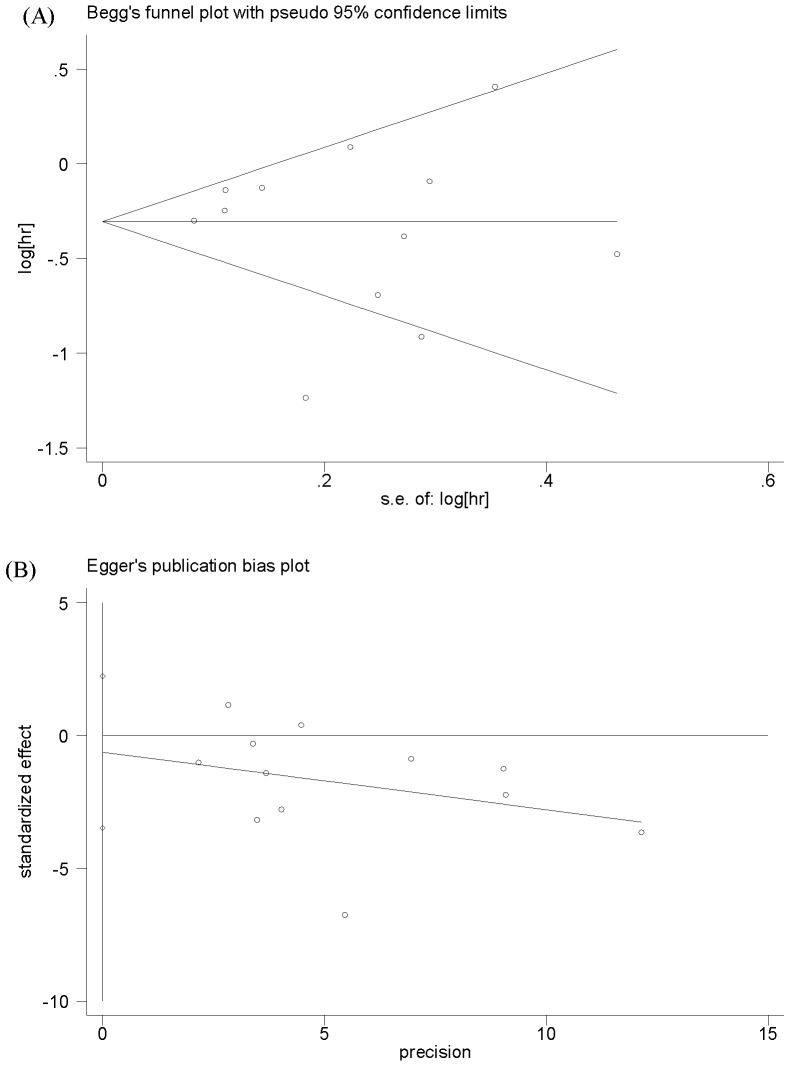
Publication bias plot for overall survival (A) Begg’s funnel plot (B) Egger’s publication bias plot.

The main results of this meta-analysis and the heterogeneity test are presented in Table 2. Among the 12 studies eligible for pooling of OS data, 7 studies provided estimated HR associated with its 95%CI [8], [10]–[14], [16]. In the remaining studies, these data points were calculated from data presented [9], [15], [17] or reconstructed from survival curve [18], [19]. Figure 2 shows the forest plot of HR for OS from each study. The pooled HR for OS in GC patients was 0.71 (95%CI: 0.57–0.87; *P* = 0.001), with significant evidence of heterogeneity between the contributing studies (*P*<0.0001). The funnel plot of HR showed no evidence of publication bias from either Begg’s test (*P* = 0.999) or Egger’s test (*P* = 0.634), which was shown in Figure 3.

**Table 2:** Meta-analysis of *H. pylori* infection with the prognosis of gastric cancer.

Stratified analysis		No. of Studies	Test of association					Test of heterogeneity	
			*Pooled HR* ^△^ *(95%CI)*	*Z*	*P-value*	*Model*	*χ^2^*	*P-value*	*I^2^(%)*
OS	Overall	12	0.71(0.57–0.87	3.27	0.001	R	44.79	<0.0001	75.4
Ethnicity									
Asian		7	0.77(0.54–1.10)	1.46	0.145	R	36.08	<0.0001	83.4
Caucasian		4	0.66 (0.50–0.87)	2.95	0.003	R	8.39	0.039	64.3
Statistical methodology									
Univariate analysis results		6	0.80(0.72–0.90)	3.88	<0.0001	F	6.76	0.239	26.1
Multivariate analysis results		6	0.56(0.37–0.86)	2.65	0.008	R	31.46	<0.0001	84.1
* H. pylori* Evaluation Method									
Serologic detection		6	0.73(0.64–0.83)	4.92	<0.0001	F	9.10	0.105	45.0
Histological analysis		6	0.60(0.42–0.85)	2.82	0.005	R	29.74	<0.0001	83.2
PCR		3	0.86(0.41–1.81)	0.40	0.690	R	10.69	0.005	81.3
Quality assessment									
High quality		10	0.66(0.52–0.85)	3.31	0.001	R	40.20	<0.0001	77.6
Low quality		2	0.99(0.54–1.84)	0.02	0.980	R	3.12	0.077	67.9
DFS	Overall	3	0.60(0.30–1.18)	1.48	0.139	R	8.00	0.018	75.0

Abbreviations: *H. pylori*, *Helicobacter pylori*; OS, overall survival; DFS, disease-free survival; PCR, Polymerase chain reaction; HR, hazard ratio; CI, confidence interval; R, random-effects model; F, fixed-effects model.

Δ HR = 1 for negative *H. pylori* status.

When assessing *H. pylori* infection on DFS in GC patients, only three studies presented data valuable for analysis [10], . The pooled HR was 0.60 (95%CI: 0.30–1.18; *P* = 0.139), with evidence of study heterogeneity (*P* = 0.018).

### Subgroup and Sensitivity Analyses

Subgroup and sensitivity analyses were further performed to evaluate the effect of *H. pylori* infection on OS in GC patients. Statistically significant heterogeneity was observed in all the subgroup analyses except for the subgroup analysis of univariate results. The results of Begg’s test and Egger’s test showed no evidence of publication bias for all subgroup analyses.

When stratified by ethnicity, the subgroup analysis in Asians yielded a HR of 0.77 (95%CI: 0.54–1.10; *P* = 0.145), whereas the subgroup analysis in Caucasians yielded a HR of 0.66 (95%CI: 0.50–0.87; *P* = 0.003).

When we stratified the studies by statistical methodology (univariate analysis results versus multivariate analysis results), the pooled HR for the univariate analysis results was 0.80 (95%CI: 0.72–0.90; *P*<0.0001); similarly, the pooled HR for the multivariate analysis results was 0.56 (95%CI: 0.37–0.86; *P* = 0.008).

When we stratified the studies by *H. pylori* evaluation method, the HR for the 6 studies using serologic detection method was 0.73 (95%CI: 0.64–0.83; *P*<0.0001), the HR for the 6 studies using histological analysis method was 0.60 (95%CI: 0.42–0.85; *P* = 0.005) and the HR for the 3 studies using polymerase chain reaction (PCR) method was 0.86 (95%CI: 0.41–1.81; *P* = 0.690).

The result was in accordance with the overall analysis when analyses were restricted to 10 high-quality studies (HR: 0.66; 95%CI: 0.52–0.85; *P* = 0.001). In contrast, the effect was not significant when analyses were restricted to 2 low-quality studies (HR: 0.99; 95%CI: 0.54–1.84; *P* = 0.980).

Sensitivity analyses showed that the HR and 95%CI did not alter substantially by removing any one study, ranged from a low of 0.68 (95%CI: 0.55–0.84; *P*<0.0001) to a high of 0.78 (95%CI: 0.68–0.90; *P* = 0.001) via omission of the study by Chen et al. [15] and the study by Kang et al. [16], respectively.

## Discussion

Meta-analysis was originally developed to combine the results of randomized controlled trials. Nowadays, this approach has been widely applied for identification of prognostic indicators in patients with malignant diseases [35], [36]. The reports about the prognostic signification of *H. pylori* infection in GC were controversial, thus the combination of data to reach a reasonable conclusion is necessary. As far as we know, this is the first meta-analysis to investigate the association between *H. pylori* infection and the prognosis of GC. Findings from the current meta-analysis suggest that positive *H. pylori* status is associated with better OS in GC patients, which may provide a new light of therapeutic and prophylactic targets in *H. pylori*-related GC.

When stratified by ethnicity, the protective role of *H. pylori* infection in the prognosis of GC was identified in subgroup analysis of Caucasians. In contrast, there was no association between *H. pylori* infection and patient survival in subgroup analysis of Asians. So far, reasons for ethnic differences remain unclear. Population differences of genetic factors, dietary behavior, environmental exposures and other factors may help explain part of the ethnic differences in patient survival with GC. Furthermore, more and larger studies in Asians, Caucasians as well as Africans are warranted in the future. The method used for the assessment of *H. pylori* status differed among these studies. In order to minimize the effects resulting from *H. pylori* evaluation methods, we investigated the effects of *H. pylori* infection on survival in three categorized groups: serologic detection group, histological analysis group and PCR group. We observed improved survival among patients with positive *H. pylori* status in both serologic detection group and histological analysis group, consistent with the overall analysis result. With regard to the statistical methodology, the results of the meta-analysis suggested an association between positive *H. pylori* status and better survival in either a univariate setting or a multivariate setting. Thus, even after adjustment for conventional prognostic factors of survival, the association observed in the univariable analysis seemed still hold in the multivariable analysis. Moreover, the significant protective effect of *H. pylori* on patient survival with GC was still observed even after excluding low quality studies or in sensitivity analysis. No improvements in terms of DFS were observed in the present meta-analysis. This result should be interpreted with caution due to the small number of contributing studies.

There is continued controversy with regard to whether *H. pylori* infection can lead to improved outcomes for GC patients. *H. pylori* is thought to be an important pathogen for GC, which indirectly promote carcinogenesis through induction of chronic inflammatory states. Once cancer has developed, persistent infection with *H. pylori* and infiltration with some leucocyte subsets seem to correlate with a favorable prognosis in *H. pylori*-related GC patients [37]. This seems paradoxical but might have a biological basis. The plausible explanations and theoretical bases may be elucidated as follows. Microbe-induced inflammation might modulate antitumor immunity. The presence of *H. pylori* acts as an adjuvant for the induction of the cellular immune response which displays a type-1 T-helper-cell (Th1) type, and a local B-cell response in gastric mucosa [38], [39]. Wherever, the relation between inflammation-related immune response and antitumor activity still needs further evidences. If further related basic experiments confirm the hypothesis, *H. pylori* might contribute to an improved antitumor immune response. Microsatellite instability may also play certain role in *H. pylori* positive GC. Microsatellite instability is a hallmark of the DNA mismatch repair deficiency that is one of the pathways of gastric carcinogenesis. Microsatellite alterations were related with a higher rate of *H. pylori* infection and a better postoperative survival [40], [41].

Despite considerable efforts to explore the possible association between *H. pylori* infection and the prognosis of GC, some limitations should be addressed. Firstly, significant between-study heterogeneity was detected in overall and subgroup analyses, which may be distorting the meta-analysis. There is no common threshold value to assign *H. pylori* status. That might account for part of the heterogeneities of all analyses. Other factors, such as ethnicity, study design and patient selection, may also be possible explanations for the heterogeneities across the studies. In this case, the random-effect model, which took heterogeneity into account, was used to analyze the studies with heterogeneity. Additionally, we did sensitivity testing and found that the HR and 95%CI did not alter substantially after removing any one study. Secondly, in the manuscript, we only discussed the protective effect of *H. pylori* for patients with GC. Other strong carcinogens and hereditary factors may contribute to the tumorigenesis of GC with non-*H. pylori* infection. The interactions between these factors and *H. pylori* infection should be elucidated in further studies. Thirdly, the secondary outcome of interest was DFS. Lacking sufficient eligible studies limited our further stratified analysis on DFS. Fourthly, only a few prospective studies were included in this meta-analysis [10], [11], [16], [19]. We have performed a subgroup analysis for the 4 prospective studies. The pooled HR was 0.38 (95%CI: 0.29–0.48; *P*<0.0001) for OS, consistent with the overall analysis result.

In conclusion, our results suggest a protective role for *H. pylori* infection in the prognosis of GC. More large-scale and well-designed prospective cohort studies from various ethnic populations are necessary to validate our findings in the future. The underlying mechanisms need to be further elucidated, which could provide new therapeutic approaches for GC.

## Supporting Information

Table S1 **PRISMA checklist.**
(DOC)

Table S2 **Methodologic quality of studies included in the meta-analysis.**
(DOC)
