# Supplementary material for: Protective Role of Helicobacter pylori Infection in Prognosis of Gastric Cancer: Evidence from 2454 Patients with Gastric Cancer
Source: PLoS One. 2013 May 7;8(5):e62440. doi: 10.1371/journal.pone.0062440 (PMC3646839; doi:10.1371/journal.pone.0062440)
Supplement: Table S2 — Methodologic quality of studies included in the meta-analysis. (DOC) [file pone.0062440.s002.doc]

| Table S2. Methodologic quality of studies included in the meta-analysis | | | | | | | | | |
| --- | --- | --- | --- | --- | --- | --- | --- | --- | --- |
| Study | Representativeness of the exposed cohort | Selection of the unexposed cohort | Ascertainment of exposure | Outcome of interest not present at start of study | Control for important factor or additional factor^a^ | Outcome assessment | Follow-up long enough for outcomes to occur^b^ | Adequacy of follow-up of cohorts^c^ | Total quality scores |
| Lee et al.[8] | ☆ | ☆ | ☆ | — | ☆☆ | ☆ | — | ☆ | 7 |
| Kurtenkov et al.[9] | ☆ | ☆ | ☆ | ☆ | ☆ | ☆ | — | — | 6 |
| Meimarakis et al.[10] | ☆ | ☆ | ☆ | ☆ | ☆☆ | ☆ | — | ☆ | 8 |
| Marrelli et al.[11] | ☆ | ☆ | ☆ | ☆ | ☆☆ | ☆ | ☆ | ☆ | 9 |
| Qiu et al.[12] | ☆ | ☆ | ☆ | ☆ | ☆☆ | ☆ | — | ☆ | 8 |
| Gan et al.[13] | ☆ | ☆ | ☆ | ☆ | ☆☆ | ☆ | — | ☆ | 8 |
| Santos et al.[14] | ☆ | ☆ | ☆ | ☆ | ☆☆ | ☆ | ☆ | — | 8 |
| Chen et al.[15] | ☆ | ☆ | ☆ | ☆ | — | ☆ | — | — | 5 |
| Kang et al.[16] | ☆ | ☆ | ☆ | ☆ | ☆☆ | ☆ | ☆ | — | 8 |
| Syrios et al.[17] | ☆ | ☆ | ☆ | ☆ | — | ☆ | ☆ | ☆ | 7 |
| Choi et al.[18] | ☆ | ☆ | ☆ | ☆ | — | ☆ | — | — | 5 |
| Hur et al.[19] | ☆ | ☆ | ☆ | ☆ | ☆☆ | ☆ | — | — | 7 |
| ^a^ A maximum of two stars can be awarded for this item. Studies controlling for stage received one star while studies controlling for other important confounders such as sex, age, grade et al. received an additional star.  ^b^A cohort study with a follow-up time ≥ 5 years or long enough for outcomes to occur was assigned one star.  ^c^A cohort study with a follow-up rate more than 75% was assigned one star. | | | | | | | | | |
